# Childhood emotional abuse and cyberbullying perpetration among Chinese university students: The chain mediating effects of self-esteem and problematic social media use

**DOI:** 10.3389/fpsyg.2022.1036128

**Published:** 2022-12-01

**Authors:** Wei Xu, Shujie Zheng

**Affiliations:** ^1^School of Educational Science, Ludong University, Yantai, China; ^2^Institute for Education and Treatment of Problematic Youth, Ludong University, Yantai, China; ^3^School of Humanities and Social Sciences, Binzhou Medical University, Yantai, China

**Keywords:** childhood emotional abuse, cyberbullying perpetration, self-esteem, problematic social media use, university students

## Abstract

Childhood abuse has been shown to have a range of adverse physical and psychological consequences, including aggression and bullying. While researchers have explored the relationship between childhood abuse and cyberbullying, little is known about the impact of emotional abuse on cyberbullying. This study examined the link between childhood emotional abuse (CEA) and cyberbullying perpetration among university students in the Chinese cultural context, as well as the chain mediating effect of self-esteem and Problematic Social Media Use (PSMU). A total of 835 university students (18–25 years old; 293 males, 542 females; *M*_age_ = 19.44 years, SD = 1.28) completed the Childhood Trauma Questionnaire Short-Form (CTQ-SF), Rosenberg Self-Esteem Scale (RSES), the Social Media Use Questionnaire (SMUQ), and Cyberbullying Inventory (CBI). The results showed that CEA and PSMU were positively correlated with cyberbullying; self-esteem was negatively correlated with cyberbullying. Besides, self-esteem and PSMU sequentially mediated the relationship between CEA and cyberbullying perpetration. The findings indicate that childhood emotional abuse may lower self-esteem and cause problematic social media use, which increases cyberbullying perpetration.

## Introduction

By the end of 2021, the number of Chinese Internet users was 1.032 billion, of which the Internet penetration rate reached 73%, and the proportion of online surfing with mobile phones was up to 99.7% [China Internet Network Information Center (CNNIC), 2022]. With the popularity of the Internet, cyberbullying has become a global phenomenon and an increasingly severe social issue. Cyberbullying perpetration is usually defined as “using electronic forms of contact to repeatedly and intentionally harm a victim who cannot defend him or herself.” (Smith et al., 2008), including various behaviors, such as harassment, denigration, masquerade, flaming, and cyberstalking (Smith, 2015). Cyberbullying differs from traditional school bullying in its anonymity, fast spreading, and uncontrollability, which may cause more critical consequences and adverse psychophysiological effects on victims.

The prevalence of cyberbullying varies in different studies due to different definitions and measurement methods (Brochado et al., 2017; Kowalski et al., 2019; Chen and Chen, 2020; Barlett et al., 2021; Eyuboglu et al., 2021; Zhu et al., 2021; Fulantelli et al., 2022). A systematic review showed that the prevalence of cyberbullying victimization among East Asian adolescents ranged from 5.8 to 56.8%, and the rate of bullying was lower than victimization (Park, M. S. et al., 2021). A study in EU member countries showed that the rate of victimization ranged from 2.8 to 31.5%, from 3.0 to 30.6% of cyber perpetration (Henares-Montiel et al., 2022). Several studies have revealed that cyberbullying may lead to anxiety, depression, loneliness, social withdrawal, substance abuse, self-harm, and suicidality (Kwan et al., 2020; Giumetti et al., 2021; Chu et al., 2022; Coelho et al., 2022; Pichel et al., 2022). However, much previous research focused on teenagers, and relatively few studies were conducted on university students. Therefore, this study will explore the influencing factors and internal mechanisms of university students’ cyberbullying perpetration to provide empirical support for scientific intervention.

Childhood emotional abuse (CEA) occurs independently of other types of childhood maltreatment (Glaser, 2002) and refers to non-physical, long-term, and harmful interactions between caregivers and children, including verbal aggression, humiliation, blaming, demeaning, or other behaviors (Glaser, 2011; Li et al., 2022). It is noticeable that childhood emotional abuse, which is more devastating than physical and sexual abuse and has potentially lifelong adverse effects, is often overlooked because there is no physical evidence of it (Rees, 2010; Dye, 2020). Studies in different countries and regions have shown high rates of CEA (Chandraratne et al., 2018; Prino et al., 2018). In a recent survey from Hong Kong, 43.3% of participants reported they had suffered emotional abuse during childhood (Fung et al., 2020).

Childhood emotional abuse is a risk factor for aggressive behavior (Wang et al., 2019a). According to the cycle of violence hypothesis, chronically abused victims in childhood are at greater risk of violence in adolescence and early adulthood (Wright et al., 2019) and are more likely to bully peers (Wang et al., 2017; Xiao et al., 2021; Park, J. et al., 2021). Recent evidence suggests that childhood abuse can significantly positively predict cyberbullying (Wang et al., 2019b; Jin et al., 2020; Fang et al., 2022; Li et al., 2022). Two recent studies in Western society have revealed a strong link between CEA and high cyberbullying perpetration among adolescents (Kircaburun et al., 2019; Emirtekin et al., 2020).

Based on previous studies, we consider that CEA is closely related to cyberbullying among university students. However, not all individuals who have suffered from domestic violence will exhibit violent behaviors. Further research is needed to elucidate the mediating or moderating mechanisms in the above relationships.

According to the self-system model (Connell and Wellborn, 1991; Skinner and Wellborn, 1994; Sandler, 2001), self-model is self-representation, which refers to the positive and negative evaluation of individuals. Typical self-evaluations include self-esteem and self-efficacy. Self-esteem refers to an individual’s positive or negative evaluations of the global perception of self. Childhood adversity, including family maltreatment, can lead to severe disruption of self-system processes (e.g., self-esteem). From the perspective of attachment theory (Riggs and Kaminski, 2010), self-esteem generates from integrating the attitudes and evaluations of others (e.g., parents and other caregivers) toward the self. Children who grow up in an abusive environment do not get the necessary support and affirmation to develop a sense of self-worth.

Previous studies have also confirmed the negative correlation between childhood abuse and self-esteem (Arslan, 2016; Lim and Lee, 2017; Luo et al., 2020; Chen et al., 2022). CEA, a part of childhood abuse, can also cause low self-esteem (Chen and Qin, 2020), which may lead to a range of emotional and behavioral problems, resulting in psychological and social maladjustment (Arslan, 2016; Lim and Lee, 2017). Several studies have reported that low self-esteem is associated with high cyberbullying (Brewer and Kerslake, 2015; Fan et al., 2019; Yang et al., 2021).

Recent evidence has demonstrated the mediating effect of self-esteem on the relationship between adversity and problem behavior in children and adolescents. We believe that inappropriate parenting (e.g., CEA) will lead to low self-esteem, and low self-esteem may exhibit aggressive behaviors to avoid inferiority and shame caused by failure (Tracy and Robins, 2003).

Social media are fertile ground for cyberbullying perpetration (Chan et al., 2021). Some researchers have mentioned that Twitter and Facebook are two main social media platforms with the highest occurrence of online bullying (Whittaker and Kowalski, 2015). Problematic Social Media Use (PSMU) refers to an over concern and powerful motivation to engage in social media (Andreassen et al., 2016), relevant signs include overuse on social media platforms, log in or check social software frequently, irritability, or anxiety when not being able to access social media (Chen et al., 2020). Numerous research has indicated that PSMU is an important predictor of online aggression and cyberbullying (Görzig and Frumkin, 2013; Barlett et al., 2018; Craig et al., 2020; Marengo et al., 2021; Borraccino et al., 2022; Giumetti and Kowalski, 2022).

Problematic Social Media Use is influenced by many factors. The Interaction of Person-Affect-Cognition-Execution (I-PACE) model explains that problematic Internet use (e.g., social media addiction) is the result of the interaction of a person, affect, cognition, and execution (Brand et al., 2016, 2019). Specifically, a person’s core characteristics are the susceptibility variables to behavior addiction, including bio-psychological factors (such as genes and early adverse experiences), psychopathological correlates (depression, anxiety, and attention deficit hyperactivity disorder), and personality traits (e.g., high impulsivity, low self-esteem, and high neuroticism). Existing studies have confirmed the link between high abuse and PSMU (Worsley et al., 2018; Kircaburun et al., 2020), as well as low self-esteem and PSMU (Saiphoo et al., 2020; Schivinski et al., 2020).

Early traumatic experiences can be internalized in the evaluation of oneself and others, which could cause damage to self-esteem. Moreover, people with lower self-esteem are sensitive to interpersonal relationships and depend on social network to establish social relationships, leading to the intemperate use of social media and ultimately increasing the risk of cyberbullying. Therefore, high emotional abuse may lead to low self-esteem and PSMU in undergraduates, which in turn causes cyberbullying perpetration.

Given all the above, the current study aims to construct a chain mediation model to test the effects of self-esteem and PSMU. Based on the existing theories and empirical research, we put forward four specific hypotheses:

*H1*: CEA would positively predict cyberbullying perpetration among university students.

*H2*: Self-esteem plays a mediating role in the relationship between CEA and cyberbullying perpetration.

*H3*: PSMU might be a mediator in the relationship between CEA and cyberbullying perpetration.

*H4*: Self-esteem and PSMU together play a chain mediating role in the relationship between CEA and cyberbullying perpetration.

## Materials and methods

### Participants

Using the cluster random sampling method, 865 students from a university in Shandong province were selected as the research objects. 30 invalid questionnaires were removed, and the remaining 835 questionnaires were valid. Among the participants, there were 293 (35.1%) male students and 542 (64.9%) female students. 416 (49.8%) were freshmen, 246 (29.5%) were sophomores, and 173 (20.7%) were juniors. 308(36.9%) came from an urban area and 527(63.1%) came from a rural area. The age ranged from 18 to 25 years (*M* = 19.44, SD = 1.28).

### Measures

#### Childhood emotional abuse

The Childhood Trauma Questionnaire Short-Form (CTQ-SF) developed by Bernstein et al. (1997, 2003) and revised by Zhao et al. (2005), measures abuse and neglect before the age of 16 years old. It includes five subscales: physical abuse (PA), emotional abuse (EA), sexual abuse (SA), emotional neglect (EN), and physical neglect (PN). Each subscale consists of five items, plus three validity items for a total of 28 items. We used the EA subscale (Chen et al., 2016; Li et al., 2020). The CTQ-SF is scored on a five-point scale (1 = never; 5 = always), with higher scores indicating higher levels of EA. The Cronbach’s α of EA in the current study was 0.70.

#### Self-esteem

The Rosenberg Self-Esteem Scale (RSES; Rosenberg, 1965; Wang et al., 1999) was used to measure the level of self-esteem. It consists of 10 items, each item is rated from 1 (strongly disagree) to 4 (strongly agree). Higher scores indicate higher levels of self-esteem. In this research, the Cronbach’s α was 0.90.

#### Problematic social media use

The Social Media Use Questionnaire (SMUQ), developed by Xanidis and Brignell (2016), assesses the problematic use of social media. The SMUQ has nine items and consists of two dimensions: withdrawal and compulsion. Participants rated each item on a five-point scale (0 = never; 4 = always). Cronbach’s α was 0.80 in the current study.

#### Cyberbullying perpetration

Cyberbullying Inventory (CBI; Erdur-Baker and Kavsut, 2007) was used to test the level of cyberbullying perpetration. The CBI has 18 items. Participants rated each item on a four-point scale (1 = never happened; 2 = happened once or twice; 3 = happened 3–5 times; and 4 = happened more than five times). Higher scores indicate serious state of cyberbullying perpetration. In this study, Cronbach’s α was 0.80.

### Procedure and statistical analysis

We choose students’ self-study time to conduct a collective test of the class. To ensure data quality, the first author, familiar with the study, served as the experimenter for each class. The study was approved by the Ethics Committee of Binzhou Medical University. To control the common method bias (CMB), we used procedural remedies and statistical remedies. The questionnaire included some reverse scoring items. Besides, the participants were told that the survey would be conducted anonymously, only for scientific research, and voluntarily.

In addition, we used a statistical test—the Harman single factor test to analyze the common method bias. The results showed that there were 10 factors with eigenvalues >1, and the first factor accounted for 16.12% variance, which is less than the 40% threshold. SPSS 26.0 and SPSS macro PROCESS model 6 (Hayes, 2013) were used for correlation analysis and chain intermediary effect test.

## Results

### Correlation analysis among variables

The mean, standard deviation, and correlation matrix of each variable are shown in Table 1. The results showed that CEA (*r* = 0.23, *p* < 0.01), PSMU (*r* = 0.20, *p* < 0.01), and cyberbullying were significantly positively correlated, while self-esteem and cyberbullying were negatively correlated (*r* = −0.13, *p* < 0.01). Additionally, there was a significant positive correlation between CEA and PSMU (*r* = 0.23, *p* < 0.01), and a significant negative correlation between CEA and self-esteem (*r* = −0.28, *p* < 0.01) and PSMU and self-esteem (*r* = −0.21, *p* < 0.01).

**Table 1:** Descriptive statistics and correlations among variables.

	*M*	*SD*	1	2	3	4
1. Childhood emotional abuse	6.92	2.57	—			
2. Self-esteem	31.94	5.28	−0.28^**^	—		
3. Problematic social media use	15.78	5.67	0.23^**^	−0.21^**^	—	
4. Cyberbullying	19.52	3.14	0.23^**^	−0.13^**^	0.20^**^	—

** *p* < 0.01.

### Mediation of self-esteem and problematic social media Use

**Figure 1:**
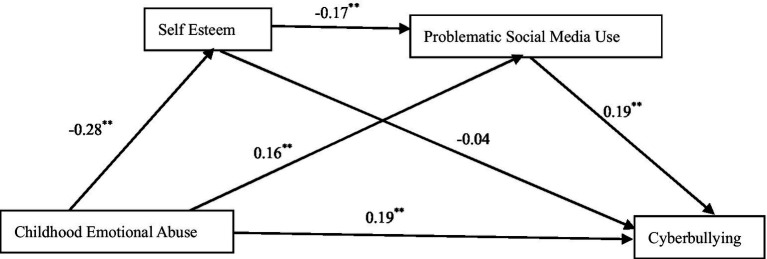
Model of the chained mediating effect of self-esteem and problematic social media use. **p < 0.01.

Using PROCESS Model 6 to test the chain mediation effect. CEA was used as a predictor variable, cyberbullying as a dependent variable, self-esteem and PSMU as mediating variables, and gender as a control variable. As shown in Figure 1, CEA negatively predicted self-esteem (*β* = −0.28, *p* < 0.01) and positively predicted PSMU (*β* = 0.16, *p* < 0.01) and cyberbullying (*β* = 0.19, *p* < 0.01). Self-esteem negatively predicted PSMU (*β* = −0.17, *p* < 0.01), but it had no significant predictive effect on cyberbullying (*β* = −0.04, *p* > 0.05). PSMU positively predicted cyberbullying (*β* = 0.19, *p* < 0.01). The results of Bootstrap mediation test are shown in Table 2. The mediating effect of self-esteem is not significant, and the 95% CI [−0.01, 0.04] includes 0. The mediating effect of PSMU was significant, with 95% CI [0.02, 0.06], and the mediating effect accounted for 12.2% of the total effect. The chain mediating effect of self-esteem and PSMU was also significant, with 95% CI [0.00, 0.02], and the mediating effect accounted for 3.72% of the total effect.

**Table 2:** Indirect effect of self-esteem and problematic social media use.

	Effect	SE	LLCI	ULCI
Total	0.06	0.02	0.03	0.10
CEA → SES → CB	0.01	0.01	−0.01	0.04
CEA → PSMU → CB	0.04	0.01	0.02	0.06
CEA → SES → PSMU → CB	0.01	0.00	0.00	0.02

## Discussion

Although the effect of childhood maltreatment on cyberbullying has acquired considerable empirical support (Wang et al., 2019b; Jin et al., 2020; Fang et al., 2022; Li et al., 2022), less is known about the potential mediating mechanisms. Based on a sample of 835 Chinese university students, we constructed a chain mediated model, and the results proved the chain mediating effect of self-esteem and social media use in the above relationship.

As expected (H1), CEA was positively associated with cyberbullying perpetration, which is consistent with the results of previous studies (Kircaburun et al., 2019; Emirtekin et al., 2020; Li et al., 2022). Undergraduates with emotional maltreated experiences are more susceptible to bullying others online. The results support the cycle of violence hypothesis that violence in adulthood can be explained by early abuse. Children who live with violent parents for a long time will inherit violent ways to solve problems, creating a terrible cycle of violence. Based on the results of this study and previous studies, we believe that CEA is a critical risk factor for cyberbullying perpetration.

Attachment theory demonstrates that children constantly insulted, terrorized, disparaged, and despised by parents will believe they are worthless, unloved, and unlovable, which seriously affects their formation of correct self-cognition and results in lower self-evaluation. The results showed a negative effect of CEA on self-esteem, which also accords with the earlier observations (Arslan, 2016; Chen et al., 2022). However, when PSMU was added, the predictive effect of self-esteem on cyberbullying was no longer significant, and the path of CEA → self-esteem → cyberbullying was not significant, which is inconsistent with hypothesis 2. This result is similar to previous studies (Xin et al., 2007; Cao and Zhang, 2018). When other variables are added, the direct predictive effect of self-esteem on aggressive behavior becomes no longer significant. Low self-esteem caused by CEA does not necessarily trigger online bullying, though, in the most simple binary relation, our result showed the negative relationship between self-esteem and cyberbullying, but in the background of multivariate context and within the group of university students, the relationship between the above two is likely to be influenced by the third variable, this study also proved it.

In line with Hypothesis 3, PSMU mediated the relationship between CEA and cyberbullying perpetration. Emotional abuse can significantly and positively predict problematic Internet use and social media use, consistent with existing research findings (Dalbudak et al., 2014; Schimmenti et al., 2014; Worsley et al., 2018; Kircaburun et al., 2020). Dalbudak et al. (2014) have found that the types of childhood abuse associated with increasing risk of Internet addiction were emotional abuse, emotional neglect, and physical neglect, among which the emotional abuse was the most important predictor. In addition, Schimmenti et al. (2014) revealed that childhood sexual abuse was associated with a sevenfold increasing risk of problematic Internet use in adolescence. This may be a strategy for individuals who suffered adversity in childhood to cope with early adverse experiences through virtual worlds (Worsley et al., 2018). Frequent and problematic use of social media increases the likelihood of witnessing and imitating online attacks, causing negative consequences of cyberbullying perpetration and victimization. However, PSMU is more strongly linked with cyberbullying perpetration (Craig et al., 2020). Thus, the mediating effect of PSMU was significant.

The current results also indicated that self-esteem and PSMU had a chain mediating effect on the relationship between emotional abuse and cyberbullying. CEA sequentially influences self-esteem and PSMU and eventually leads to cyberbullying among university students. Individuals exposed to parental maltreatment in childhood suffer from emotional trauma and unsatisfied psychological needs. They constantly receive and accumulate negative emotions and feedback from caregivers and gradually internalize negative evaluations of themselves with low self-worth and self-esteem. Moreover, to overcome the trauma caused by early adverse experiences, children and adolescents seek their positive values in social networks to obtain emotional satisfaction, which leads to the excessive use of social media and increases the chances of hurting others and being hurt by others. How does CEA influence cyberbullying among university students? Some researchers suggest that it is through dark personality traits (Kircaburun et al., 2019), while others consider it is through the chain mediating effect of hostile attribution bias and anger rumination (Li et al., 2022). Our results explain why CEA is associated with cyberbullying in terms of self-esteem and PSMU, supporting the I-PACE model and enriching our understanding of the relationship between early adverse experiences and cyberbullying perpetration.

This study confirms that CEA sequentially influences cyberbullying through self-esteem and PSMU in a Chinese cultural context. In the Chinese sample, low self-esteem caused by CEA does not necessarily lead to cyberbullying. It is the overuse of social media that results in the risk of cyberbullying. Whether this conclusion is applicable to other cultures remains to be further tested.

## Limitations and future directions

Several limitations of the current study should be noted. Firstly, the cross-sectional design cannot provide evidence for causal relationships among variables, and longitudinal studies should be carried out in the future, which would be more conducive to understanding the impact of emotional abuse on cyberbullying. Secondly, self-report measures were used to collect data, while retrospective self-report is prone to biases. In addition, the sensitivity of abuse or cyberbullying is easily affected by social desirability bias. Thirdly, the samples were taken from undergraduates in China, and cyberbullying has cultural differences (Craig et al., 2020), so future research should conduct cross-cultural tests.

## Data availability statement

The raw data supporting the conclusions of this article will be made available by the authors, without undue reservation.

## Ethics statement

The studies involving human participants were reviewed and approved by Ethics Committee of Binzhou Medical University. The patients/participants provided their written informed consent to participate in this study.

## Author contributions

WX conducted the survey, analyzed the data, and wrote the manuscript. SZ designed the study and revised the manuscript. All authors contributed to the article and approved the submitted version.

## Funding

The work was supported by the Social Science Popularization and Application Research Project of Shandong Province (2022-SKZZ-02).

## Conflict of interest

The authors declare that the research was conducted in the absence of any commercial or financial relationships that could be construed as a potential conflict of interest.

## Publisher’s note

All claims expressed in this article are solely those of the authors and do not necessarily represent those of their affiliated organizations, or those of the publisher, the editors and the reviewers. Any product that may be evaluated in this article, or claim that may be made by its manufacturer, is not guaranteed or endorsed by the publisher.
